# Polyphenol Characterization and Skin-Preserving Properties of Hydroalcoholic Flower Extract from *Himantoglossum robertianum* (Orchidaceae)

**DOI:** 10.3390/plants8110502

**Published:** 2019-11-14

**Authors:** Miriam Bazzicalupo, Bruno Burlando, Marcella Denaro, Davide Barreca, Domenico Trombetta, Antonella Smeriglio, Laura Cornara

**Affiliations:** 1 Department of Earth, Environment and Life Sciences, University of Genova, 16132 Genova, Italy; miriamb91@hotmail.it; 2 Department of Pharmacy, University of Genova, 16132 Genova, Italy; burlando@difar.unige.it; 3 Institute of Biophysics, National Research Council (CNR), 16149 Rome, Italy; 4 Department of Chemical, Biological, Pharmaceutical and Environmental Sciences, University of Messina, 98168 Messina, Italy; mdenaro@unime.it (M.D.); dbarreca@unime.it (D.B.); dtrombetta@unime.it (D.T.); asmeriglio@unime.it (A.S.)

**Keywords:** antioxidants, collagenase, elastase, flavonoids, keratinocytes, skin aging, *Himantoglossum robertianum*

## Abstract

*Himantoglossum robertianum* (Loisel.) P. Delforge is a Mediterranean orchid whose propagation in vitro has been achieved, making it eligible as a source of bioactive substances. Flowers were analyzed by light and SEM microscopy and used to obtain a polyphenol-rich, hydroalcoholic flower extract (HFE). HFE was characterized for total phenols, flavonoids and proanthocyanidins, and for polyphenol profile by RP-LC-DAD. Antioxidant assays, in vitro collagenase and elastase inhibition, and MTT and cell motility assays on HaCaT keratinocytes were done. Microscopy showed epidermal cells containing anthocyanins in the flower labellum. Flavonoids (flavones and flavan-3-ols) represented the most abundant compounds (42.91%), followed by scopoletin (33.79%), and phenolic acids (23.3%). Antioxidant assays showed strong activities, rating ORAC > FRAP > TEAC > β-carotene bleaching > DPPH > iron-chelation. Biological assays showed elastase and collagenase inhibition (up to 42% and 78%, respectively), improvement of HaCaT cell viability after treatment with 500 μM H_2_O_2_ (from 30% to 84% of control), and stimulation of cell migration rate up to 210% of control. In summary, HFE counteracted different free radicals, while protective properties were shown by cell-free and cell-based bioassays, suggesting the possible use of *H. robertianum* flowers for skin-preserving, repair, and anti-aging applications.

## 1. Introduction

Orchids have been widely used in the folk tradition to treat several ailments, including tuberculosis, inflammation, hepatitis, wounds and sores, tumor, asthma, malaria, and menstrual disorders [1]. The uses of *Anacamptis morio* L. and *Epipactis helleborine* (L.) Crantz for wound healing have been reported [2], while a number of species are known as sources of bioactive compounds [3,4,5].

Different studies have started to explore the biological activities of orchid extracts, especially with reference to skin care applications. A cosmetic serum containing 5% orchid extracts, including the Marcella Koss intergeneric hybrid of *Brassocattleya*, has been tested in vivo for its skin-whitening properties on melasma and lentigo senilis in Japanese women [6]. In a study about the potential of *Vanda coerulea* Griff. ex Lindl., and *V. teres* (Roxb.) Lindl. extracts as an anti-aging and skin-moisturizing agent, the importance of mucilage to maintain skin hydration has been highlighted [7]. 

Extracts from various portions of three hybrids of *Phalaenopsis* species, bearing white, yellow, and purple pigmentation, have been tested for their antioxidant power. This study has revealed that in each species, flower and leaf extracts have higher antioxidant activities than pedicel and root extracts, due to higher levels of flavonoids and anthocyanins, especially in the purple species [8]. It has also been demonstrated that the Ayurvedic formulation Asthvarga, containing orchids as main component, has strong antioxidant capability, preventing DNA damage from oxidative stress [9]. Following these scientific achievements, the industrial exploitation of orchids for skin care purposes has been started. However, few phytochemical studies have been carried out on the inflorescences of European Orchidaceae [10,11].

We chose to investigate *Himantoglossum robertianum* (Loisel.) P. Delforge (syn. *Barlia robertiana*) due to its large inflorescences, and because it can be easily propagated in vitro, making it exploitable as a source of bioactive substances without damaging natural populations [12]. *H*. *robertianum* is a Mediterranean orchid with scattered occurrence and large distribution area, spanning from Portugal and Morocco to Anatolia. Typical habitats include poor grassland, garrigue, scrub, and open woodland. The plant grows up to an altitude of 1700 m, and blooms from January to April. It is called ‘giant orchid’, as it exceeds most European wild orchids by its large size, reaching a height of 50–80 cm [13]. The species is included in the Appendix II of the Convention on International Trade in Endangered Species of Wild Fauna and Flora (CITES), and as Least Concern on the French red list (UICN France et al. 2010). In Italy it is a protected entity at national level and is covered by total regional protection in different areas (https://www.actaplantarum.org/ipfi/floraz_base_map_prot.php?s=1147). However, despite its protected status, the species has been found to expand its presence across the Mediterranean distributional range [14].

In Turkey, the species is cultivated as ornamental plants in kitchen gardens [15], while in rural communities around Catania (Italy), it is roasted and eaten [16]. Similar uses are reported for the related species *H. hircinum* (L.) Spreng. (syn. *H. affine*), *H. jankae* Somlyay, Kreutz & Óvári, and *H. comperianum* (Steven) P. Delforge, which in Iran are harvested for salep flour production, have a role in folk medicine, and their tubers are used for producing beverage and ice-cream [17].

*H*. *robertianum* has been studied for its essential oil [18], but there is little information on its phytochemistry and biological properties. Therefore, the aim of our study was to investigate the polyphenol composition and biological properties of flowers, in order to verify potentials for dermatological and cosmetic uses. The study included flower micro-morphological characterization, and the extraction of a polyphenolic fraction that was used for phytochemical investigations, in vitro cell-free experiments of enzyme inhibition, and the in vitro treatment of keratinocytes. 

## 2. Results

### 2.1. Flower Morphological Characterization

**Figure 1:**
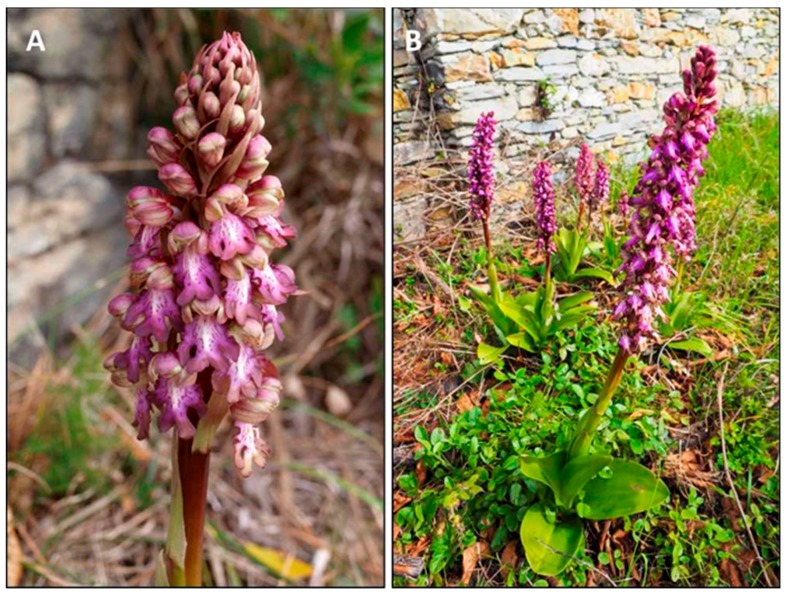
(**A**) Flower spike of *H. robertianum*. (**B**) Plant habitus in olive grove environment.

The strongly scented flowers are arranged in dense spikes with 25–40 large flowers each, growing up to 15–30 cm (Figure 1A,B). Flowers are variable in color, from pink-purple to greenish-white, show an upper hood made up of 3 petals, and a large lower lip with a lighter spotted center and darker lip margins. The lip measures about 2.0 cm, is divided into three lobes, and the central lobe is further subdivided. Considering color variability, we collected flowers showing a rather homogeneous purplish color in the labellum and sepals.

**Figure 2:**
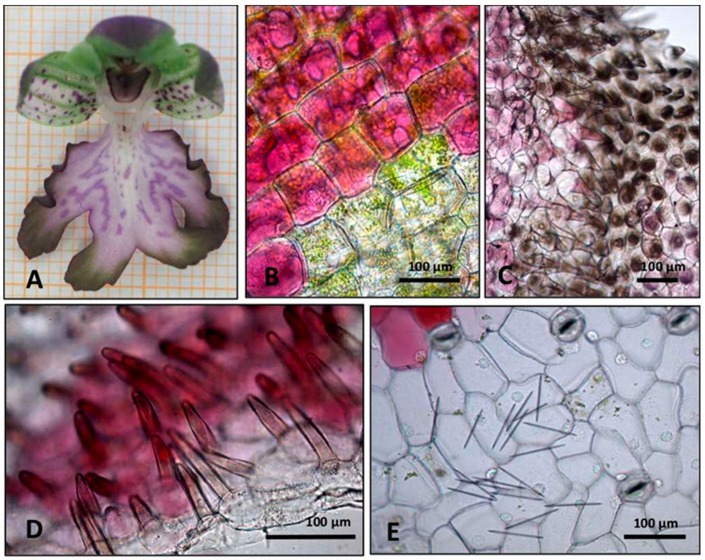
Stereomicroscope (**A**) and light microscope (**B**–**E**) pictures of the flower. (**A**) Total view showing three sepals, two petals, and a labellum. (**B**) Central portion of the labellum, showing purple, anthocyanin-rich cells interspersed among unpigmented cells. (**C**) Short papillose cells in an invagination of the medium-high portion of the labellum lateral arm. (**D**) Elongated pigmented papillae in the sub-stigmatic zone of the central labellum. (**E**) Stomata and raphides in the sepal.

**Figure 3:**
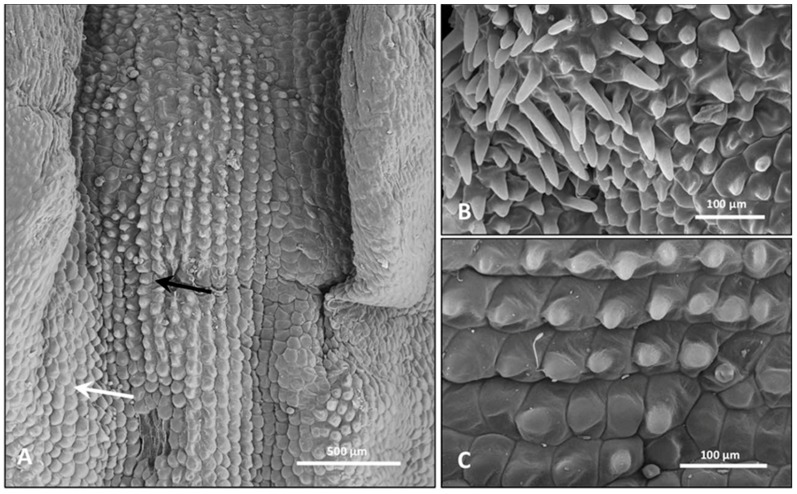
SEM micrographs of flower portions. (**A**) Overview of the central and lateral regions (arm) of the labellum in which two kinds of papillae are observed. Most prominent papillae were found in the central zone of the labellum (black arrow), while in the lateral zone the papillae become increasingly more flattened (white arrow). (**B**) Elongated papillae in the substigmatic zone of the central labellum. (**C**) Magnified view of the labellum arm where gradual flattening of papillose cells is visible.

Flower samples observed under stereomicroscope showed three sepals and petals covering the fertile gynostemium (Figure 2A). The central petal, or labellum, differs from the lateral ones in color, shape, and size. It is composed by a median central region and two lateral zones (arms), showing epidermal cells with different colors and shapes (Figure 2A, Figure 3A). The labellum forms the spur that is probably involved in deceptive pollination, acting as a resting place for pollinators and as an osmophore involved in the emission of attractive odorants. 

In sections observed under light microscopy, epidermal cells of the central region of the labellum appeared purple-red due to the presence of anthocyanins (Figure 2B). In the lateral region of the labellum, several short papillose cells, brown-purple in color, were found (Figure 2C) among more flattened, sub-polygonal cells covered by waxes (Figure 2C, Figure 3C). The sub-stigmatic zone of the central labellum showed outgrowths made of long papillose cells (Figure 2D, Figure 3B), possibly facilitating the emission of volatile compounds. Raphides and stomata were observed both in the sepals and in the labellum (Figure 2E).

### 2.2. Phytochemical Characterization

By ultrasound-assisted method, we obtained a hydroalcoholic flower extract (HFE) with a high extraction yield (8.28%). A preliminary phytochemical screening (Table 1) revealed high total phenol content (243.7 mg GAE/100 g fresh weight, FW), with flavonoids representing the most abundant compounds (398.1 ± 9.8 mg QE/100 g FW), and a lower presence of anthocyanins (4.89 mg ChE/100 g FW). The higher content of flavan-3-ols determined by vanillin index (3.31 ± 0.032 mg CatE/100 g FW), with respect to proanthocyanidins (0.05 ± 0.001 mg CyE/100 g FW), suggests the presence of polymeric compounds. This assumption was corroborated by the high value of the polymerization index (vanillin index/proanthocyanidin content ratio), yielding a rough estimate of the polyphenol polymerization degree. 

**Figure 4:**
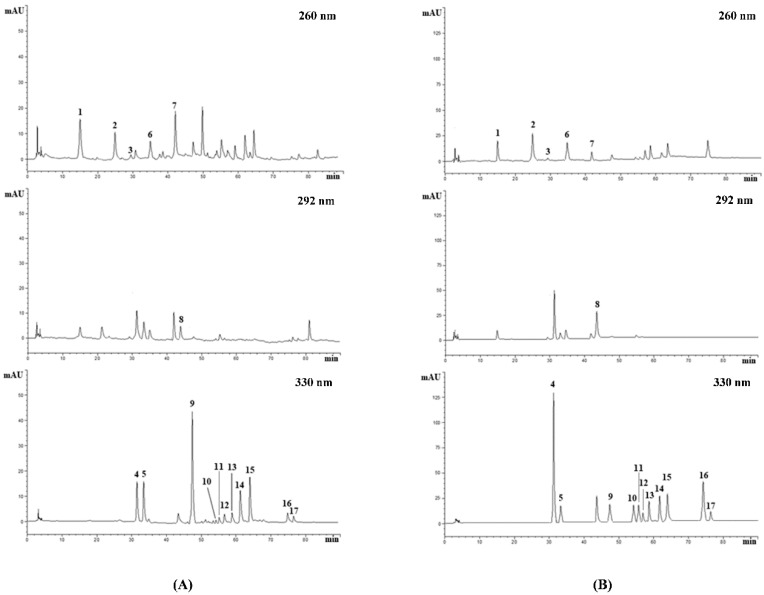
Representative RP-LC-DAD chromatogram of HFE (Panel **A**) and reference standard mix 10 μg/mL (Panel **B**), acquired at 260, 292, and 330 nm. Peak numbers correspond to compounds listed in Tables 2 and 4.

The RP-LC-DAD analysis confirmed what was observed in the preliminary phytochemical screening, allowing us to identify and quantify 17 compounds, with a total polyphenol content of 144.5 mg/100 g FW (Figure 4). 

Among polyphenols, flavonoids represent the most abundant compounds (42.91%), followed by scopoletin, the only coumarin identified (33.79%), and phenolic acids (23.3%). Interestingly, flavonoids belong to only two subclasses, i.e., flavones (79.85%) and flavan-3-ols (20.15%). The most abundant flavonoid was kaempferol-3-*O*-rutinoside (21.1 mg/100 g FW), while among phenolic acids, the most abundant was caffeic acid (11.5 g/100 FW) (Table 2).

### 2.3. Antioxidant and Free-Radical Scavenging Activity

In order to evaluate the antioxidant and free-radical scavenging activity of HFE, various in vitro cell-free assays, based on different environments and reaction mechanisms, were carried out. All assays showed dose-dependent (R2 ≥ 0.99) antioxidant and free-radical scavenging activities, with the following order of potency ORAC > FRAP > TEAC > β-carotene bleaching > DPPH > Iron-chelating activity (Table 3). These data indicate that HFE can counteract different charged free-radical types, especially peroxyl ones.

### 2.4. Effects of HFE on Keratinocytes and Skin Enzymes

Effects of HFE on cell viability were preventively tested by the MTT assay, finding IC_05_ > 500 and IC_50_ > 1000 μg/mL. Therefore, in order to keep HFE at subtoxic levels, all experiments were conducted by using 500 μg/mL as the highest concentration.

**Figure 5:**
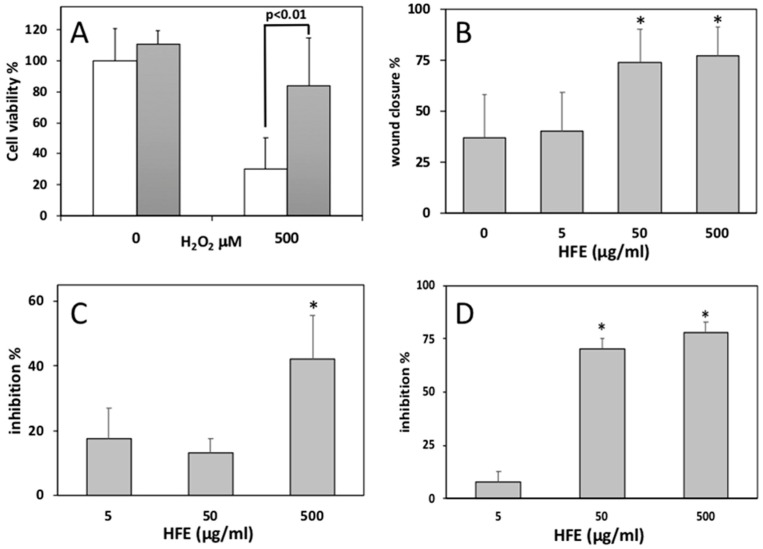
Biological activities of HFE. All data are expressed as means ± S.D. (**A**) Protective effect of pre-incubation with 500 μg/mL HFE on cell viability reduction induced by 500 μM H_2_O_2_, evaluated by MTT assay on HaCaT keratinocytes. Data are formazan absorbance at 570 nm standardized as percent of control (*n* = 8). Differences between means have been evaluated by t test. (**B**) Increased wound closure induced by HFE in a scratch wound healing assay conducted in vitro on HaCaT keratinocytes. Data are wound closures measured at 24 h since wounding, and expressed as percent of total closure (*n* = 30–200). * = *p* < 0.01 according to Bonferroni test. (**C**) In vitro inhibition of porcine pancreas elastase by 500 μg/mL HFE. Data are percent inhibition obtained from absorbances at 410 nm (*n* = 3 independent measures). Statistical comparisons as in B. (**D**) In vitro inhibition of *Clostridium histolyticum* collagenase by HFE. Data are percent inhibition obtained from enzyme kinetics derived from absorbances at 345 nm, at the time points of 0 and 15 min (*n* = 3 independent measures). Statistical comparisons as in B.

In order to evaluate the antioxidant potential of HFE directly on cells, we pre-exposed HaCaT cells to HFE for 24 h, and then to HFE combined with H_2_O_2_ for a further 24 h. Controls were run by incubating cells with HFE alone for 48 h, or with H_2_O_2_ alone for 24 h. At the end of incubations, cell viability was assessed by MTT assay. The complex of data showed that 500 μg/mL HFE induced a significant protection against the injurious effects of 500 μM H_2_O_2_ (Figure 5A). 

The scratch wound assay, conducted for 48 h on HaCaT cells, showed a significant increase of cell migration after incubation with 50 and 500 μg/mL HFE (Figure 5B). 

Finally, we performed cell-free assays to verify the ability of HFE to inhibit two major enzymes involved in skin extracellular matrix degradation, viz. elastase and collagenase. In the elastase test, a significant average inhibition of 42% was found with 500 μg/mL HFE (Figure 5C), while in the collagenase test significant average inhibitions of 70% and 78% were obtained with 50 and 500 μg/mL HFE, respectively (Figure 5D). 

## 3. Discussion

The polyphenol characterization of HFE showed that the extract is very rich in flavonoids, scopoletin, and phenolic acids. It is well known that the antioxidant and free-radical scavenging properties of polyphenols are directly correlated with the number of hydroxyl groups linked to the phenolic structure, and indirectly correlated with their glycosylation degree [19]. Flavones are the most abundant sub-class of flavonoids in HFE, but they are mostly glycosylated, and therefore, they should only marginally contribute to the observed antioxidant and free-radical scavenging activities. In contrast, flavan-3-ols like catechin and epicatechin, also having catechol structure, could play a primary role in antioxidant activities, together with major polyhydroxylated phenolic acids like protocatecuic, chlorogenic, and caffeic acids. 

Scopoletin, as previously demonstrated, showed a good scavenging ability against ABTS^•+^, but was not effective against other charged radicals, while it showed only a weak antioxidant activity. Also, in coumarins the catechol group markedly contributes to the antioxidant activity, while glycosylation adversely affects it. In addition, the α-pyrone ring makes coumarins more hydrophobic than phenolic acids, and therefore more reactive with respect to lipid peroxidation [20]. In conclusion, the complex of phytochemicals suggests that HFE can act as a powerful scavenger of different charged radicals. 

In parallel with phytochemical characterization and antioxidant evaluation, skin-preserving properties have been clearly suggested by the complex of our bioassays. Significant protection of keratinocytes against injurious effects has been found after double exposure to HFE and H_2_O_2_ as the oxidant agent, but only at high HFE doses. The lack of a pan-antioxidant protection could reflect the known tendency of some polyphenols, e.g., flavonoids, to elicit pro-oxidant activities under specific redox conditions [21,22]. However, polyphenols are also widely known to exert antioxidant effects, as fully confirmed in this study by the panel of specific assays. Hence, the polyphenols of *H. robertianum*, used at proper doses, could support skin redox balance processes, thus contributing to prevent or counteract oxidative-related dysfunctions, especially those leading to skin ageing. This kind of activity should be further potentiated by stimulation of keratinocyte mobilization, revealed by our scratch wound assay, suggesting a support to skin repair following injury or degenerative processes.

The elastase inhibition induced by HFE rates at average levels among plant extracts, while collagenase inhibition approaches topmost values obtained with polyphenol-rich white tea extract [23]. Collagen and elastin are the most abundant protein constituents of the dermal extracellular matrix, being primarily affected by photoaging and other oxidative processes that cause wrinkles and reduction in skin thickness [24]. Hence, the results obtained with HFE on matrix degrading enzymes make this orchid-derived product a very promising ingredient for skin aging remedies.

Orchids are in general protected plant species, but the development of protocols for in vitro propagation is making these plants exploitable for industrial purposes without affecting wild populations [25]. These techniques have been applied also to *H. robertianum*, through protoplast isolation and in vitro propagation from asymbiotic seed germination [26,27]. Hence, the giant orchid *H. robertianum* shows remarkable biological properties of its flower extract and can be exploited in environment-friendly mode, suggesting its possible use for post-traumatic and post-inflammatory skin repair, antiaging treatments, and skin preserving applications.

## 4. Materials and Methods

### 4.1. Plant Materials

Flowers of *H*. *robertianum* were gathered by LC and MB from wild populations growing at Taggia (43°52’05.2” N, 7°50’14.6” E), and Carpasio (43°57’24.8” N, 7°50’38.7” E) (Imperia, Liguria, Italy) from February to May 2018. Sampling of the species, protected under law, was allowed by the Ligurian Region Government with act n. 363/29-01-2018. Only flowers were carefully sampled from resident plants, in order to minimize damage to populations. The species has a quite distinctive habitus and size, and it was determined in the field by LC and MB, and two voucher specimens (one from each sampling site) were deposited at the Herbarium of DISTAV, University of Genova, Genova, Italy (number: GE 1038).

### 4.2. Reagents and Cells

All reagents were purchased from Sigma Aldrich (Milan, Italy), unless otherwise indicated. The HaCaT human keratinocyte cell line was purchased from the Biological Bank of the Azienda Ospedaliera Universitaria San Martino-IST, Genova, Italy. Cells were cultured at 37 °C, in a 5% CO_2_, humidified atmosphere, using Dulbecco’s Modified Eagle Medium (DMEM, EuroClone, Milan, Italy) enriched with 10% (*v/v*) FBS, 1% glutamine, and 1% antibiotic.

### 4.3. Light and Scanning Electron Microscopy Analyses

Flower portions were observed by a Leica M205C stereomicroscope, coupled with EC3 camera and LAS EZ V1.6.0 image analysis software. Epidermal peels of the fresh flowers were mounted on glass slides and observed by a Leica DM 2000 transmission-light microscope coupled with a computer- driven DFC 320 camera (Leica Microsystems, Wetzlar, Germany).

For Scanning Electron Microscope (SEM) analyses, small flower pieces were fixed in Finefix working solution (Milestone s.r.l., Bergamo, Italy) with 70% ethanol, incubated overnight at 4 °C, dehydrated by ethanol series (70–100%) at 60 min intervals, and dried using a Critical Point Dryer Processor (K850CPD 2M Strumenti S.r.l., Roma, Italy) [28]. Dried samples were positioned on aluminum stubs, covered with 10nm gold particles, and observed in a VEGA3-Tescan-type LMU microscope (Tescan Orsay Holding, a.s., Brno, Czech Republic), at an accelerating voltage of 20 kV.

### 4.4. Hydroalcoholic Flower Extract (HFE) Preparation

Flowers were gently grounded by a blade mill (IKA^®^ A11 basic analytical mill) under liquid nitrogen. One hundred milliliters of 70% ethanol were added to 10 g of powdered sample mixing for 3 min. The extraction was carried out three times by sonication in an ice-cold bath for 5 min using a 3 mm titanium probe, set to 200W power and 30% amplitude signal (Vibra CellTM Sonics Materials inc., Danbury, CT, USA). Thereafter, the sample was centrifuged at 3000 rpm for 15 min (NEYA 10R, REMI, Carpi, Italy) and the supernatant was collected and evaporated by rotary evaporator (BUCHI R-205, Cornaredo, Italy). Dry HFE was dissolved in 70% ethanol in order to obtain a 50 mg/mL stock solution, which was properly diluted to carry out polyphenol characterization, antioxidant assays, cell-free bioassays, and cell treatments. 

### 4.5. Chemical Characterization

#### 4.5.1. Total Phenols

Total phenols content was established by Folin–Ciocalteu assay as previously reported [29]. The absorbance was recorded at 786 nm using an UV-Vis spectrophotometer (Shimadzu UV-1601, Kyoto, Japan). Results were expressed as mg of gallic acid equivalents (GAE)/100 g of sample fresh weight (FW).

#### 4.5.2. Flavonoids

Total flavonoids content of HFE (0.625–10 mg/mL) was determined colorimetrically using an UV-Vis Spectrophotometer (Shimadzu UV-1601) as previously reported [19]. Quercetin (0.125–1.0 mg/mL) was used as reference compound. Flavonoid content was expressed as mg of quercetin equivalents (QE)/100 g of sample FW.

#### 4.5.3. Vanillin Index

The proanthocyanidin and anthocyanin contents were determined as previously reported [30], recording the absorbance of sample by an UV–Vis spectrophotometer (Shimadzu UV-1601, Kyoto, Japan) at 500 nm against a blank. Catechin was used as reference compound (0–500 μg/mL) and results were expressed as mg of catechin equivalent (CatE)/100 mg of sample FW.

#### 4.5.4. Proanthocyanindin and Anthocyanin Content

The proanthocyanidin content was determined by an UV–Vis spectrophotometer (Shimadzu UV-1601, Kyoto, Japan), as previously reported [30,31]. Results were expressed as mg of Cyanidin chloride equivalents (CyE)/100 g of sample FW and Chrysanthemin (Cyanidin-3-*O*-glucoside) chloride equivalents (ChE)/100 g of sample FW, respectively.

#### 4.5.5. Polyphenol Profile by Reversed Phase Liquid Chromatography with Diode Array Detection (RP-LC-DAD)

The qualitative and quantitative determination of polyphenols in HFE was carried out as previously reported [32]. An Agilent high performance liquid chromatography system (1100 series, Santa Clara, CA, USA), equipped with a photodiode-array detector (DAD, G1315), was used. Briefly, the chromatographic separation was carried out by a 5 µm, 250 mm × 4.6 mm ODS3 Prodigy column (Phenomenex, Torrance, CA, USA), maintained at 25 °C, with solvent A (water/acetic acid, 97:3, *v/v*) and solvent B (methanol). The gradient elution was: 0–3 min, 0%B; 3–9 min, 3% B; 9–24 min, 12% B; 24–30 min, 20% B; 30–33 min, 20% B; 33–43 min, 30% B; 43–63 min, 50% B; 63–66 min, 50% B; 66–76 min, 60% B; 76–81 min, 60% B; 81–86 min, 0% B and equilibrated 4 min for a total run time of 90 min. Flow rate and injection volume was 1.0 mL/min and 50 µL, respectively. UV-Vis spectra of polyphenols were recorded from 190 to 400 nm. Peak identity was confirmed by comparing the retention time and the absorption spectra with those of pure (≥ 99%) commercially available standards (Extrasynthese, Genay, France). Chromatograms were acquired at 260 and 292 nm for phenolic acids and flavan-3-ols, and at 330 nm for flavones and coumarins. Quantitative analysis was carried out using external calibration curves of reference compounds (concentration range 0.1–20 μg/mL) (Table 4).

### 4.6. Antioxidant and Free-Radical Scavenging Activities

#### 4.6.1. Scavenging Activity of 2,2-diphenyl-1-picrylhydrazyl (DPPH) Free Radical

The DPPH free radical scavenging activity of HFE (31.25–500 μg/mL) was evaluated using an UV-Vis Spectrophotometer (Shimadzu UV-1601, Kyoto, Japan), as previously reported [19]. The results were expressed as inhibition (%) of the radical activity calculating the half-maximal inhibitory concentration (IC_50_) with the respective confidence limits (C.L.) at 95%.

#### 4.6.2. Trolox Equivalent Antioxidant Capacity (TEAC)

The antioxidant activity against 2,2’-azino-bis(3-ethylbenzothiazoline-6-sulphonic acid) (ABTS) radical was carried out as previously reported [33]. Fifty microliters of HFE (6.25–100 μg/mL) were added into 1 mL of TEAC reagent and incubated in dark at RT for 6 min. Absorbance was recorded at 734 nm with an UV-Vis Spectrophotometer (Shimadzu UV-1601, Kyoto, Japan). Results were expressed as inhibition (%) of the radical activity calculating the half-maximal inhibitory concentration (IC_50_) with the respective C.L. at 95%.

#### 4.6.3. Ferric-Reducing Antioxidant Potential (FRAP)

The free-radical scavenging capacity against 2,4,6-tris(2-pyridyl)-s-triazine (TPTZ) radical was performed according to a previous study [33]. Briefly, 25μL of HFE (1.25–20 μg/mL) was added to 1.5 mL of daily fresh FRAP reagent pre-warmed at 37 °C, and incubated for 4 min at RT. The absorbance was recorded at 593 nm by an UV-VIS Spectrophotometer (Shimadzu UV-1601, Kyoto, Japan), and results were expressed as inhibition (%) of the radical activity calculating the half-maximal inhibitory concentration (IC_50_) with the respective C.L. at 95%.

#### 4.6.4. Oxygen Radical Absorbance Capacity (ORAC)

ORAC was evaluated as previously reported [19]. Briefly, 20 μL of HFE (0.3125–10.0 μg/mL) diluted in 75 mM phosphate buffer solution (pH 7.4) was mixed with 120 μL of fresh daily 117 nM fluorescein solution. After 15 min incubation at 37 °C, 60 μL of fresh daily AAPH solution (40 mM) was added. The fluorescence was monitored every 30 s for 90 min (λ_ex_ 485 nm; λ_em_ 520 nm) using a fluorescence plate reader (Fluostar Omega, BMGLabtech, Ortenberg, Germany). Phosphate buffer was used as negative control while trolox was used as reference standard (10–100 μM). Results were expressed as inhibition (%) of the radical activity calculating the half-maximal inhibitory concentration (IC_50_) with the respective C.L. at 95%.

#### 4.6.5. β-Carotene Bleaching Assay

The β-carotene bleaching assay was performed as previously described [19]. Aliquots of the fresh β-carotene emulsion (8.0 mL) were mixed with 320μL of HFE solutions (12.50–200 µg/mL). An emulsion without β-carotene was used as negative control. The absorbance was recorded at 470 nm at the starting time (t = 0), and then incubated at 50 °C in a water bath for 120 min, recording the absorbance every 20 min. Butylated-hydroxytoluene (BHT) 1 mg/mL was used as positive control. The antioxidant activity was expressed as inhibition (%) of the β-carotene bleaching calculating the half-maximal inhibitory concentration (IC_50_) with the respective C.L. at 95%. 

#### 4.6.6. Iron-Chelating Activity

The iron-chelating activity of HFE was evaluated according to a previous study [34], with some modifications. Briefly, 50 μL of FeCl_2_∙4H_2_O solution (2.0 mM) was added to 100 μL of HFE (62.5–100 μg/mL) incubating at RT for 5 min. After that, 100 μL of ferrozine solution (5 mM) was added to the reaction mixture and the sample solution diluted to 3 mL with deionized water, mixed and incubated for 10 min at RT. The absorbance was read at 562 nm using an UV-VIS Spectrophotometer (Shimadzu UV-1601, Kyoto, Japan). Results were expressed as inhibition (%) of the Fe^2+^ chelating capacity calculating the half-maximal inhibitory concentration (IC_50_) with the respective C.L. at 95%.

### 4.7. Biological Assays

#### 4.7.1. Collagenase and Elastase Inhibition Assays

The elastase and collagenase assays were performed according to a previously-reported method [35]. Briefly, elastase (EC 3.4.21.36) inhibition was evaluated in a reaction mixture containing 200 mM tris(hydroxymethyl)aminomethane (TRIS) pH 8.0, 10 mM N-succinyl-Ala-Ala-Ala-p-nitroanilide (Sigma-Aldrich, cat. S4760), 4 units/mL of elastase from porcine pancreas (Sigma-Aldrich, E1250), and serial water dilutions of HFE stock solutions in 30% ethanol (final concentrations: 5, 50, 500 μg/mL). After incubation at RT for 10 min, plates were read at 410 nm on a Varian Cary-50 Bio spectrophotometer (Agilent, Milan, Italy). 

Collagenase (EC 3.4.24.3) inhibition was assayed in a mix containing 0.16 units/mL of collagenase from *Clostridium histolyticum* (Sigma-Aldrich, cat. MAK293B), 20% N-[3-(2-furyl)acryloyl]-L-leucyl-glycyl-L-prolyl-L-alanine (FALGPA, substrate, MAK293C), 74% collagenase assay buffer (MAK293A), and water dilutions of HFE stock solution in 60% ethanol, to reach the indicated concentrations. Plates were read at 345 nm, at 37 °C, for 15 min in kinetic mode.

#### 4.7.2. Cytotoxicity and Cytoprotection Assays

Effects of HFE on cell viability were assessed by the 3-(4,5-dimethylthiazol-2-yl)-2,5-diphenyltetrazolium bromide (MTT) assay. HaCaT were settled on 96-well plate for 24 h, incubated with 5, 50, or 500 μg/mL HFE for 24 h, processed for MTT staining, and read at 570 nm. Absorbance data were used to obtain dose–response curves and IC_50_ values. 

For the assay of HFE cytoprotective properties, HaCaT were settled as above in 96-wells plates, preincubated for 24 h with 5, 50, or 500 μg/mL HFE, and then co-incubated for further 24 h with the above HFE doses combined with 500, 750, or 1000 μM H_2_O_2_. Cell viability was then determined by MTT test. Results were expressed as the percentage of viable or dead cells (ratio of unstained or stained cells to the total number of cells, respectively).

#### 4.7.3. In Vitro Wound Healing Assay

The migration rate of HaCat cells exposed to HFE was assessed by the scratch wound assay method as reported by Muniandy et al. [36]. Briefly, cells were seeded on 12-well plates (2 × 10^5^ cells/well), grown to monolayer, and wounded with a 100 μL pipette tip. One well was immediately fixed in Finefix working solution and stained with TBO 0.1%, to represent the T0 sample. Negative control was exposed to medium without serum, and control to complete medium. The remaining wells were exposed for 48 h to 5, 50, or 500 μg/mL HFE. After incubation, cells were washed with PBS, fixed, and stained as above. Images of wounds were taken with a Leica M205 C stereomicroscope coupled to a Leica EZ 2.1.5 camera, and wound width was measured by image analysis with ImageJ software (https://imagej.nih.gov/ij/). Cell migration was expressed as the percentage of wound closure, calculated with the following formula:

Wound closure (%) = 100 × ((wound width at 0 h) − (wound width at 48 h))/(wound width at 0 h).(1)

### 4.8. Statistical Analysis

The statistical analysis used in this study was based on one-way analysis of variance (ANOVA) and Bonferroni post-hoc test for multiple pairwise mean comparisons. The statistically significant was considered when p < 0.01. Data were showed as mean ± S.D.

## Figures and Tables

**Table 1:** Phytochemical screening of the hydroalcoholic *Himantoglossum* flower extract (HFE).

Phytochemical Screening	HFE
Total phenols (mg GAE ^1^/100 g FW)	243.7 ± 26.2
Flavonoids (mg QuE ^2^/100 g FW)	398.1 ± 9.8
Anthocyanins (mg ChE ^3^/100 g FW)	4.89 ± 0.05
Proanthocyanidins (mg CyE ^4^/100 g FW)	0.05 ± 0.001
Vanillin index (mg CatE ^5^/100 gFW)	3.31 ± 0.03 ^6^
Polymerization index	66.2

^1^ GAE = Gallic acid equivalents; ^2^ QE = Quercetin equivalents; ^3^ ChE = Chrysanthemin equivalents; ^4^ CyE = Cyanidin chloride equivalents; ^5^ CatE = Catechin equivalents. ^6^ Data are expressed as means ± standard deviation (S.D.) of three independent experiments.

**Table 2:** HFE polyphenol profile.

Peak n. ^1^	Compound	R_t_^2^ (min)	λ_max_ (nm)	mg/100 g FW ^3^
*Phenolic acids*				
1	Protocatecuic acid	15.057	260; 294	7.8 ± 0.06
2	Hydroxybenzoic acid	25.543	255	0.09 ± 0.001
4	Chlorogenic acid	31.399	294; 326	10.85 ± 0.44
5	Caffeic acid	33.614	232; 323	11.52 ± 0.37
6	Vanillic acid	35.252	260; 292	0.08 ± 0.002
8	Coumaric acid	43.811	233; 310	3.33 ± 0.02
*Flavan-3-ols*				
3	Catechin	29.451	234; 279	2.63 ± 0.02
7	Epicatechin	42.062	232; 280	9.87 ± 0.38
*Flavones*				
10	Isovitexin	55.300	270; 337	3.82 ± 0.04
11	Naringenin-7-*O*-glucoside	55.878	284; 340	0.98 ± 0.02
12	Vitexin	57.145	268; 338	5.47 ± 0.05
13	Rutin	59.281	256; 356	5.08 ± 0.03
14	Kaempferol-3-*O*-rutinoside	61.975	266; 348	21.1 ± 0.25
15	Roifolin	64.619	266; 338	9.23 ± 0.08
16	Luteolin	74.693	254; 350	0.86 ± 0.04
17	Apigenin	76.417	236; 338	3.01 ± 0.07
*Coumarins*				
9	Scopoletin	47.373	296; 344	48.85 ± 0.48

^1^ Peak numbers refer to Figure 4. ^2^ Rt, Retention time. ^3^ Data are expressed as mg/100 g FW, and as means ± S.D. of three independent experiments (n = 3).

**Table 3:** HFE antioxidant and free-radical scavenging activities.

Antioxidant Assay	HFE
	IC_50_^1^ μg/mL (95% C.L. ^2^)
DPPH	211.1 (181.1–245.3) ^3^
FRAP	8.85 (7.69–10.18)
TEAC	25.04 (20.41–30.71)
ORAC	2.52 (2.19–2.9)
β-carotene bleaching	31.43 (22.12–44.66)
Iron-chelating activity	440.8 (330.1–588.6)

^1^ IC_50_ = half-maximal inhibitory concentration; ^2^ C.L. = Confidence limits. ^3^ Data are expressed as means ± S.D. of three independent experiments (n = 3).

**Table 4:** Reference compounds used for quantitative analysis.

Peak n. ^1^	Compound	R_t_ (min)	λ_max_ (nm)	Regression Coefficient (R^2^)
1	Protocatecuic acid	15.055	260; 294	0.9999
2	Hydroxybenzoic acid	25.545	255	0.9997
3	Catechin	29.453	234; 279	0.9997
4	Chlorogenic acid	31.401	294; 326	0.9999
5	Caffeic acid	33.617	232; 323	0.9998
6	Vanillic acid	35.254	260; 292	0.9996
7	Epicatechin	42.065	232; 280	0.9999
8	Coumaric acid	43.813	233; 310	0.9999
9	Scopoletin	47.375	296; 344	0.9999
10	Isovitexin	55.302	270; 337	0.9997
11	Naringenin-7-*O*-glucoside	55.881	284; 340	0.9998
12	Vitexin	57.145	268; 338	0.9996
13	Rutin	59.282	256; 356	0.9997
14	Kaempferol-3-*O*-rutinoside	61.977	266; 348	0.9998
15	Roifolin	64.620	266; 338	0.9999
16	Luteolin	74.695	254; 350	0.9999
17	Apigenin	76.418	236; 338	0.9998

^1^ Peak numbers refer to Figure 4.
